# Proximal Tubule Reabsorption and CKD Progression in the General Population

**DOI:** 10.1016/j.ekir.2026.103801

**Published:** 2026-01-29

**Authors:** Marius Øvrehus, Jesse Ikeme, Ronit Katz, Knut A. Langlo, Yngvar Haaskjold, Michael G. Shlipak, Joachim H. Ix, Stein Hallan

**Affiliations:** 1 Department of Clinical and Molecular Medicine, Faculty of Medicine and Health Sciences, Norwegian University of Science and Technology, Trondheim, Norway; 2 Department of Nephrology, St. Olavs Hospital, Trondheim, Norway; 3 Department of Medicine, Kidney Health Research Collaborative of San Francisco, San Francisco Veterans Affairs Healthcare System and University of California San Francisco, San Francisco, California, USA; 4 Department of Obstetrics & Gynecology, University of Washington, Seattle, Washington, USA; 5 Division of Nephrology-Hypertension, Department of Medicine, University of California San Diego; 6 Veterans Affairs San Diego Healthcare System, San Diego, California, USA

**Keywords:** alpha-1-microglobulin, biomarker, chronic kidney disease, general population, progression, tubule functions

## Abstract

**Introduction:**

Current markers of chronic kidney disease (CKD) primarily reflect kidney glomerular health and do not include markers of kidney tubule health.

**Methods:**

We used a case-cohort design with 1246 adults randomly sampled from the general population–based Trøndelag health study (HUNT)-3 study (Norway, 2006–2008) and 445 cases experiencing major adverse kidney events (MAKE; progressive CKD [*n* = 341], rapid estimated glomerular filtration rate [eGFR] decline [*n* = 232], kidney replacement therapy [KRT, *n* = 10], or kidney death [*n* = 9]) during 13 years of follow-up. Associations of proximal tubule reabsorption markers in the urine (alpha-1-microglobulin [A1M], beta-2-microglobulin [B2M], and cystatin C [CysC]) with MAKE were evaluated using weighted logistic regression analyses.

**Results:**

At baseline, mean age was 53 years (SD: 15), eGFR was 92 ml/min per 1.73 m^2^ (SD: 22), and median urine albumin-creatinine-ratio was 1.3 mg/mmol (interquartile range [IQR]: 1.0–1.8). The prevalence of diabetes, cardiovascular disease (CVD), and treated hypertension was 5%, 9%, and 23%, respectively. Independent of eGFR, albuminuria, and CKD risk factors, each 1-SD higher urine A1M and B2M were associated with greater odds of MAKE (odds ratio [OR]: 1.45, 95% confidence interval [CI]: 1.17–1.81 and OR: 1.23, 95% CI: 1.05–1.44, respectively). No significant association was observed for urine CysC (OR: 0.94, 95% CI: 0.74–1.19). There was significant interaction between A1M and eGFR (*P* = 0.03), and 2-way sensitivity analysis displayed that levels of urine A1M particularly influenced the MAKE risk in those with eGFR of 45 to 75 ml/min per 1.73 m^2^ at baseline.

**Conclusion:**

The proximal tubule reabsorption markers, urine A1M and B2M, were associated with MAKE beyond eGFR, albuminuria, and CKD risk factors. Assessing tubule health may improve CKD staging and risk stratification.

## Graphical abstract

**Figure:**
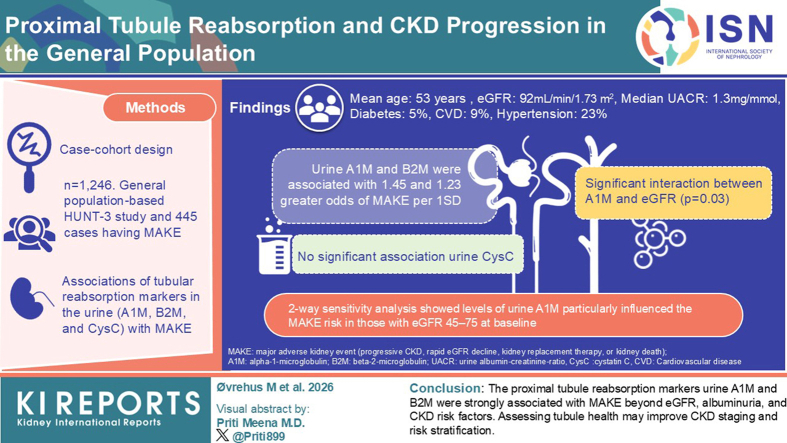

When assessed using eGFR and albuminuria, CKD is highly prevalent and imposes a heavy burden on patients and society.^1^ Glomerular dysfunction and damage have been the nephrologist’s focus for several decades, even though the kidney tubules and interstitium constitute over 90% of kidneys’ volume and are responsible for most of its homeostatic actions. Glomerulonephritis now represents a minority of CKD,^2^ whereas diabetes, hypertension, and vascular disease are the main CKD causes, potentially affecting both glomerular and tubulointerstitial structures. To address these challenges, biomarkers assessing dimensions of kidney health beyond glomerular filtration are urgently needed to advance current capabilities in quantifying risk of adverse kidney outcomes, identifying new opportunities for drug monitoring, and improving pathophysiological understanding.^3^

Proximal tubule resorption is fundamental for maintaining salt and fluid balance and for avoiding urine losses of glucose, proteins, and other nutrients. A1M is a small circulating protein produced primarily in the liver that is filtered by the glomerulus and subsequently almost completely reabsorbed by the proximal tubule in healthy individuals.^4^ Higher urinary levels of A1M, representing dysfunction of the proximal tubule, have been associated with worse kidney outcomes among adults with acute kidney injury,^5^ diabetic kidney disease,^5^ and HIV^6^; and among kidney transplant recipients.^7^ Among community-living individuals without diabetes or CKD, higher urine A1M was significantly associated with incident CKD in a meta-nalysis of the US-based ARIC, MESA, and REGARDS cohorts after adjustment for eGFR and albuminuria.^8^ A clear association was demonstrated in the ARIC cohort, whereas the association was nonsignificant after adjustment for eGFR in MESA and not demonstrated at all in the REGARDS cohort. Replication in other regions, inclusion of participants with diabetes or reduced eGFR, and testing other suggested biomarkers such as urine B2M and urine CysC are therefore needed to further explore proximal tubule reabsorption. Several other biomarkers that reflect tubular cell injury and repair processes have been studied (e.g., kidney injury molecule-1, neutrophil gelatinase-associated lipocalin, monocyte chemotactic protein-1, etc.), particularly in the context of acute kidney injury. However, biomarkers reflecting residual functional capacity, such as those indicating proximal tubular reabsorption or secretion, or the synthesis of proteins like uromodulin and epidermal growth factor, have often shown stronger prognostic value for CKD than the injury-related markers.^3^

The Kidney Disease: Improving Global Outcomes 2012 guidelines on CKD definition and classification added albuminuria as a second dimension of CKD distinct from low eGFR, and advocated for a new focus on prevention and early CKD diagnosis.^9^ However, more information is still needed on pathophysiology, diagnosis, and prognosis, especially at the early stages; and kidney biomarkers reflecting key tubule functions may be helpful by characterizing a novel, third dimension of kidney health. To that end, we evaluated the associations between urinary markers of proximal tubule reabsorption (A1M, B2M, and CysC) and MAKE in the general population using a case-cohort design.

## Methods

### Population and Study Design

HUNT is a large community-based health study in Central Norway. Starting in 1986 (HUNT-1), the entire adult population of North Trøndelag county has been invited every 10th year to participate in study visits whereby clinical measurements and biospecimens (blood and urine) are obtained. Participation rates have been very high (54%–89%), and the cohort has been regarded as representative of the Norwegian population.^10^ In addition, relevant socioeconomic, health care, and kidney-specific relationships in Norway are similar to other Western industrialized countries.^11^ All participants signed a broad consent form for use of health data, including linkage to the many high-quality health and administrative registries in Norway and information from medical records. The current study was approved by the Regional Committee for Medical Research Ethics.

**Figure 1:**
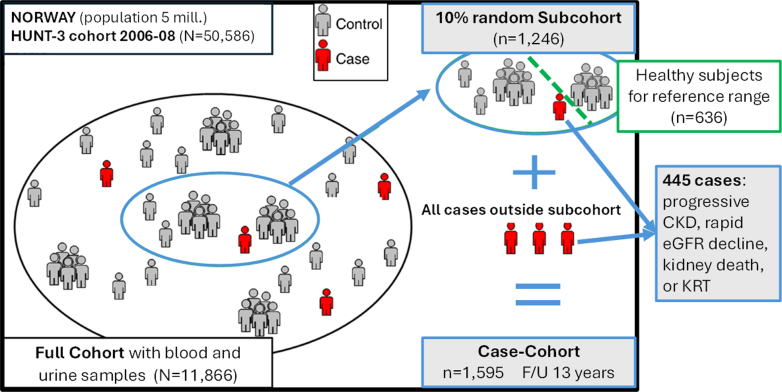
Design of case-cohort and inclusion of general population-based participants from the Norwegian HUNT 3 study. CKD, chronic kidney disease; eGFR, estimated glomerular filtration rate; F/U, follow-up; KRT, kidney replacement therapy.

We included participants from the HUNT-3 study (2006–2008) using a case-cohort design. Nonfasting blood samples were drawn in all 50,586 participants in HUNT-3, and a midstream urine sample was obtained in a subgroup of 11,866 participants.^12^ Both urine and blood samples were donated at the same time at the examination center. Urine samples were frozen within 2 hours without pH adjustment or preservatives added and stored continuously at −80 °C without thawing before being analyzed in 2023. From these 11,866 participants with complete biosamples, we selected a 10% random subcohort, which were comparable to the total HUNT-3 study for all major baseline characteristics (Supplementary Table S1). We supplemented this subcohort with the inclusion of all participants outside the subcohort who experienced our primary MAKE outcome during follow-up (Figure 1).

### Proximal Tubule Biomarker

We measured a panel of 3 low-molecular-weight proteins in the urine that are believed to reflect proximal tubule reabsorption because of their free filtration over the glomerular membrane and near-complete tubular reabsorption in healthy persons: A1M, B2M, and CysC. All measurements were conducted at the HUNT biobank and Levanger Hospital, Norway. Urine A1M was measured on a standard nephelometer (Siemens BNII, Siemens, Germany). The lower limit of detection was 5.6 mg/l, and we found a coefficient of variation of 1.8% with the high calibrator sample (37.8 mg/l) and 6.7% with the low calibrator sample (14.6 mg/l). Urine B2M and CysC were measured on a MESO QuickPlex SQ 120 multiplexing plate reader for electro-chemiluminescence immunoassays from Meso Scale Discovery (Rockwell, MD). Their coefficient of variation was 6.9% and 8.0%, respectively.

To establish valid reference ranges, we identified a healthy reference group from our random subcohort. This group included participants who reported good or excellent general health and had none of the following CK- related conditions: diabetes, CVD, treated hypertension, current smoking, body mass index > 35 kg/m^2^, eGFR < 60 ml/min per 1.73 m^2^, or urinary albumin-to-creatinine ratio (uACR) > 3 mg/mmol (30 mg/g). In line with standard clinical chemistry practice, we defined “abnormal high” biomarker levels as values > 97.5th percentile in this healthy subgroup: A1M > 22.1 mg/l, B2M > 2.1 mg/l, and CysC > 2.0 mg/l. We further categorized values in the reference range as “low-normal,” “mid-normal,” and “high-normal” based on the 60th and 80th percentiles. These cut-points were selected pragmatically: for A1M, 60% had values below the assay’s limit of detection, and the 80th percentile then effectively divides those with detectable levels into approximately equal subgroups. To facilitate comparisons of strengths of association, we applied a similar categorization strategy to urine B2M and CysC even if these biomarkers were measurable in all participants.

### Covariates

Study questionnaires were used to determine medical history. General health was reported as “bad,” “not so good,” “good,” or “very good.” Diabetes mellitus was defined as having a diabetes mellitus diagnosis made by a physician or having a nonfasting serum glucose > 11 mmol/l at the HUNT examination. CVD included previous myocardial infarction, angina pectoris or percutaneous coronary intervention, hemorrhagic and/or ischemic stroke, transient ischemic attack, and peripheral arterial disease. Smoking status was reported as “never,” “former,” or “current smoker,” and those not responding to the questions were classified as ”missing.” Body mass index was calculated as weight in kg divided by the square of height in m (kg/m^2^). Blood pressure was recorded in the sitting position using an automatic oscillometric method (Dinamap 845XT; Critikon, Tampa, FL) with 1-minute intervals between 3 standardized measurements, and the mean of the last 2 values was reported.

Serum creatinine was measured using the Jaffé method and calibrated to isotope-dilution mass-spectroscopy level using an enzymatic method (Roche). GFR was estimated using the Chronic Kidney Disease Epidemiology consortium 2009 creatinine-based formula assuming all participants were of White race.^13^ Urine albumin was determined using an immune-turbidimetric method (Abbot Park, IL), and we reported the mean of 3 samples.

### Outcome Ascertainment

Kidney function trajectory was based on serum creatinine measured at HUNT-3 examination, clinical testing by general practitioners and hospital outpatient clinics over the following 10 years, and at the HUNT-4 examination (2017–2019). The median number of GFR estimates per participant was 8 (IQR: 5–14). Participants were observed until 2020 with an observation time ranging from 0.1 to 13.8 years (median: 12.9 year, IQR: 12.6–13.3).

We used a 4-point composite MAKE defined as progressive CKD, rapid eGFR decline, KRT, or death of kidney cause.^14^ Progressive CKD was defined as an eGFR decline during follow-up of ≥ 25% compared with baseline and worsening of CKD stage.^9^ Rapid eGFR decline was defined by eGFR slope > −3 ml/min per 1.73 m^2^/yr, including ≥ 4 measurements over ≥1 year. Participants were classified as needing KRT when starting dialysis or receiving a kidney transplant, and kidney death included participants who had a distinct kidney diagnosis as their primary cause of death based on information from the Norwegian Registry of Death. All health care systems in Norway are funded by the Government, and reporting to central KRT and mortality registries are mandatory (98% coverage reported for both).^15^^,^^16^ Furthermore, information on kidney function was available for 95% of participants included in the current study.

### Statistical Analysis

Continuous variables at baseline were summarized as mean (SD) or as medians (IQR) for nonnormally distributed variables, and categorical variables were summarized as counts (%). For the longitudinal case-cohort analyses, the randomly selected participants of the subcohort were weighted by the inverse of their sampling probability (× 9.94). Cases were assigned a weight of 1, consistent with standard case-cohort design,^17^ and the estimates thereby approximate those from the full cohort. Robust variance estimation was used to account for the sampling design and give correct *P*-values and 95% CIs. Unadjusted event proportions were calculated to display overall risk across categories of the exposure variable. Because the exact timing of the MAKE composite outcome could not be determined for all components, we used logistic regression to estimate ORs, adjusting for relevant covariates, as our primary analysis. Models included adjustment for urine creatinine (to account for urine tonicity at the time of sample collection), age, sex, body mass index, smoking, hypertension, diabetes, and CVD. A final model additionally included albuminuria and eGFR to determine whether proximal tubule markers added information beyond standard clinical kidney biomarkers. Proximal tubule biomarkers were analyzed both as continuous (per 1 SD higher biomarker level) and as categorical variables, as described above. We assessed whether nonlinear transformations of the biomarker variables provided a better model fit using fractional polynomial modeling in Stata. The fractional polynomial procedure did not identify any transformations that improved fit over a simple linear specification, and therefore biomarkers were entered in their original (untransformed) form despite moderate right-skewness in their distributions.

We performed several sensitivity analyses to support our primary findings. First, we analyzed the 4 MAKE subcomponents as separate outcomes, and we used eGFR declines > 30% and 40% as secondary outcome.^18^ Second, Cox regression analysis was performed for KRT, kidney death, and with an approximated time-to-event for progressive CKD. Third, A1M values below the detection limit were imputed using truncated regression in Stata (*mi impute truncreg*) (StataCorp LLC., College Station, TX), bounded at 0 and modeled on age, sex, diabetes, blood pressure variables, CVD, uACR, CKD status, eGFR, body mass index, and urine creatinine (20 imputations). Finally, we performed subgroup analysis to explore potential effect modifications by age, sex, hypertension, diabetes, CVD, albuminuria, and eGFR on the biomarker associations with MAKE. The joint influence of biomarker levels versus eGFR with the risk of MAKE was explored graphically as a global 2-way sensitivity analysis. All analyses were conducted using Stata version 18.

## Results

After a median observation time of 13 years, 445 participants experienced a MAKE. Of these, 96 MAKEs occurred among the 1246 participants randomly sampled from the HUNT-3 cohort, and 349 occurred among other HUNT-3 participants from outside the subcohort (Figure 1). Of the 445 events, only 19 were KRT or kidney death, whereas 334 reflected progressive CKD and 92 were rapid kidney function decline. The total case-cohort had a mean age of 53 (SD: 15) years and 54% were women. Diabetes mellitus and CVD were prevalent in 5% and 9%, respectively. Mean blood pressure was 131/74 (SD 18/12) mm Hg, 23% were taking antihypertensive medications, eGFR was 92 (SD: 22) ml/min per 1.73 m^2^ and median uACR was 1.3 mg/mmol (IQR: 1.0–1.8). In Table 1, we present further details, including baseline characteristics and biomarker levels by non-MAKE and MAKE groups. The prevalence of abnormal high A1M was 5% and 18% in non-MAKE and MAKE participants, respectively (Supplementary Table S2 and Supplementary Figure S1 includes more details, including B2M, CysC, and distributions).

**Table 1:** Baseline characteristics of participants from the HUNT-3 study

Characteristics	no-MAKE	MAKE	Total
*N*	1150	445	1595
Age (yr)	50.3 (13.6)	59.3 (17.8)	52.8 (15.4)
Sex			
Female	630 (54.8%)	228 (51.2%)	858 (53.8%)
Male	520 (45.2%)	217 (48.8%)	737 (46.2%)
General health			
Bad	13 (1.2%)	11 (2.6%)	24 (1.5%)
Not so good	234 (20.8%)	127 (30.0%)	361 (23.3%)
Good	677 (60.1%)	230 (54.4%)	907 (58.6%)
Very good	202 (17.9%)	55 (13.0%)	257 (16.6%)
Smoking			
Never	486 (42.3%)	175 (39.3%)	661 (41.4%)
Former	313 (27.2%)	157 (35.3%)	470 (29.5%)
Current	230 (20.0%)	71 (16.0%)	301 (18.9%)
Missing data	121 (10.5%)	42 (9.4%)	163 (10.2%)
Physical exercise active			
No	489 (47.8%)	131 (34.3%)	620 (44.1%)
Yes	534 (52.2%)	251 (65.7%)	785 (55.9%)
BMI (kg/m^2^)	26.8 (4.2)	27.5 (4.6)	27.0 (4.3)
Systolic BP (mm Hg)	130.1 (16.8)	132.7 (19.2)	130.8 (17.5)
Diastolic BP (mm Hg)	74.5 (11.4)	73.0 (12.5)	74.1 (11.8)
Antihypertensive drugs			
No	988 (85.9%)	248 (55.7%)	1,236 (77.5%)
Yes	162 (14.1%)	197 (44.3%)	359 (22.5%)
Diabetes mellitus			
No	1,120 (97.4%)	390 (87.6%)	1,510 (94.7%)
Yes	30 (2.6%)	55 (12.4%)	85 (5.3%)
Prevalent CVD			
No	1089 (94.7%)	363 (81.6%)	1452 (91.0%)
Yes	61 (5.3%)	82 (18.4%)	143 (9.0%)
Glucose (mmol/l)	5.53 (1.22)	6.03 (2.41)	5.66 (1.64)
Cholesterol (mmol/l)	5.48 (1.13)	5.40 (1.17)	5.46 (1.14)
HDL cholesterol (mmol/l)	1.35 (0.36)	1.34 (0.37)	1.35 (0.36)
Triglycerides (mmol/l)	1.60 (0.99)	1.82 (1.03)	1.65 (1.00)
uACR (mg/mmol)	1.25 (0.96–1.63)	1.80 (1.26–2.74)	1.34 (1.00–1.86)
eGFR (ml/min per 1.73 m^2^)	99.8 (14.1)	70.5 (24.5)	92.1 (21.7)
u-Alpha-1-microglobulin (mg/l)	5.60 (5.60–9.62)	7.88 (5.60–17.8)	5.60 (5.60–11.3)
u-Beta-2-microglobulin (mg/l)	0.48 (0.22–0.79)	0.49 (0.19–0.98)	0.48 (0.21–0.82)
u-Cystatin C (mg/l)	0.21 (0.07–0.49)	0.21 (0.08–0.48)	0.21 (0.08–0.49)

BMI, body mass index; BP, blood pressure; CVD, cardiovascular disease; HDL, high-density lipoprotein; IQR, interquartile range; u-, urinary; uACR, urinary albumin-to-creatinine ratio.

Continuous data are mean (SD) or median (IQR). Categorical data are numbers (%).

**Figure 2:**
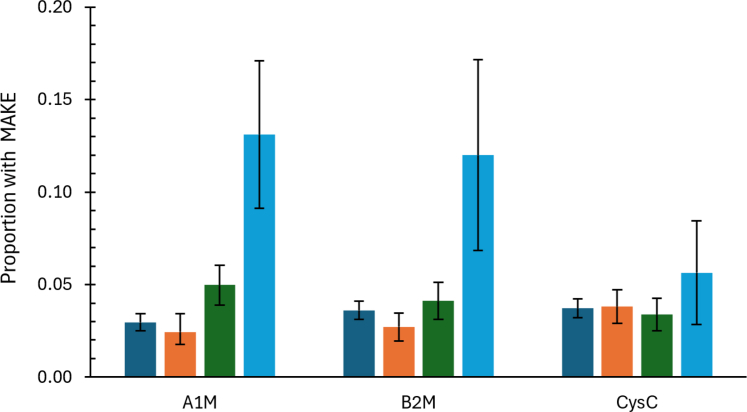
Proportion with MAKE by categories of urine biomarkers. Data show the unadjusted absolute risk with 95% confidence intervals for participants experiencing a MAKE by caegory (low-normal: 0–59th percentile in healthy subjects without chronic kidney disease risk factors; mid-normal: 60th–79th percentile; high-normal: 80th–97.5th percentile, and abnormal high: > 97.5th percentile, also among healthy subjects) for each biomarker. A1M, alpha-1-microglobulin; B2M, beta-2-microglobulin; CysC, cystatin C; MAKE, major adverse kidney events.

The proportions of participants experiencing a MAKE are displayed in Figure 2, stratified by biomarker subgroups. Participants with abnormal high levels of A1M or B2M had a 4-fold and 3-fold risk of MAKE compared with their low-normal groups, respectively; whereas only a modest elevation of risk was observed for abnormal high CysC. In regression analyses only adjusted for urine creatinine, higher concentrations of A1M and B2M were associated with higher odds of MAKE, with A1M having the strongest association (OR: 1.7 [95% CI: 1.2–2.4] per 1 SD higher A1M concentration, Table 2). For both biomarkers, the associations were only slightly attenuated when adjusted for traditional CKD risk factors and remained significant after adjustment for uACR and eGFR: OR: 1.5 [95% CI: 1.2–1.8] and OR: 1.2 [95% CI: 1.1–1.4] for A1M and B2M, respectively. For urine CysC, there was no significant association with MAKE. When biomarkers were analyzed by percentile categories, participants with abnormal high urine A1M concentration had > 2-fold odds of MAKE compared with the low-normal group in fully adjusted model (OR: 2.1, 95% CI: 1.1–4.1). Similarly, participants with abnormal high urine B2M concentrations had > 3-fold odds of MAKE when adjusting for general risk factors; however, the risk was substantially attenuated after additional adjustment for albuminuria and eGFR (OR: 1.6, 95%: 0.7–4.2). Categories of CyC were not significantly associated with MAKE.

**Table 2:** Associations of urine biomarkers of proximal tubule reabsorption with risk of MAKE over 13-year follow-up among HUNT-3 participants from the Norwegian general population

Exposure variable	Definition	MAKE / Total	Model 1 OR (95% CI)	Model 2 OR (95% CI)	Model 3 OR (95% CI)
A1M					
Per 1 SD higher		444/1589	1.74 (1.24–2.42)^a^	1.53 (1.24–1.90)^a^	1.45 (1.17–1.81)^a^
Low-normal	(< 5.6 mg/l)	193/828	1.00 (reference)	1.00 (reference)	1.00 (reference)
Mid-normal	(5.6–9.9 mg/l)	61/307	0.82 (0.59–1.13)	0.74 (0.52–1.06)	0.57 (0.32–1.02)
High-normal	(10.0–22.1 mg/l)	113/330	1.71 (1.30–2.26)^a^	1.47 (1.06–2.04)^a^	1.31 (0.85–2.02)
Abnormal high	(> 22.1 mg/l)	77/124	4.94 (3.35–7.26)^a^	3.21 (2.06–5.01)^a^	2.07 (1.05–4.05)^a^
B2M					
Per 1 SD higher		445/1595	1.43 (1.25–1.62)^a^	1.36 (1.20–1.55)^a^	1.23 (1.05–1.44)^a^
Low-normal	(< 0.6 mg/l)	259/957	1.00 (reference)	1.00 (reference)	1.00 (reference)
Mid-normal	(0.6–0.9 mg/l)	62/285	0.74 (0.54–1.02)	0.80 (0.54–1.18)	0.75 (0.37–1.51)
High-normal	(1.0–2.1 mg/l)	85/282	1.14 (0.86–1.54)	1.42 (1.03–1.97)	1.24 (0.79–1.94)
Abnormal high	(> 2.1 mg/l)	38/65	3.65 (2.19–6.07)^a^	3.42 (1.97–5.96)^a^	1.66 (0.66–4.20)
CysC					
Per 1SD higher		445/1589	1.12 (0.97–1.28)	1.11 (1.01–1.22)	0.94 (0.74–1.19)
Low-normal	(< 0.3 mg/l)	265/955	1.00 (reference)	1.00 (reference)	1.00 (reference)
Mid-normal	(0.3–0.6 mg/l)	86/304	1.02 (0.77–1.36)	0.92 (0.66–1.28)	1.25 (0.85–1.85)
High-normal	(0.7–1.9 mg/l)	71/273	0.90 (0.66–1.22)	0.90 (0.64–1.26)	0.92 (0.48–1.77)
Abnormal high	(> 2.0 mg/l)	22/57	1.54 (0.89–2.66)	1.27 (0.56–2.86)	1.07 (0.40–2.85)

A1M, alpha-1-microglobulin; B2M, beta-2-microglobulin; BMI, body mass index; CI, confidence interval; CKD, chronic kidney disease; CysC, cystatin C; eGFR, estimated glomerular filtration rate; MAKE, major adverse kidney events; OR, odds ratio.

MAKE/total are the numbers included in the full case-cohort study group, and risk is calculated using logistic regression with appropriate weights to account for oversampling of cases. Abnormal high was defined as above the 97.5th percentile in a healthy group of participants totally free for CKD risk factors, and participants with normal range values were categorized as low-normal, mid-normal, and high-normal using the 60th and 80th percentiles. Data are presented as OR with 95% CIs, and model 1 is adjusted for urine creatinine; model 2 is additionally adjusted for age, sex, systolic blood pressure, use of antihypertensive medication, BMI, smoking, diabetes mellitus, and CVD; and model 3 is adjusted for urine albumin and eGFR in addition to model 2.

a Indicate *P* < 0.05.

As sensitivity analyses, we tested whether A1M and B2M are associated with the various MAKE subcomponents (Table 3), and we performed Cox regression analyses (approximated time-to-event for the incident CKD and not available for rapid eGFR decline outcomes). Most analyses still displayed a significant association, and all showed similar direction and magnitude as our main results. Likewise, using eGFR decline > 30% and decline > 40% as outcomes, we tested indexing of urine biomarkers to urine creatinine and urine osmolality, and we imputed A1M below limit of detection with only minor differences from our main results (Supplementary Tables S3–S5, respectively).

**Table 3:** Sensitivity analysis of the association between tubule reabsorption markers and the various subcomponents of our MAKE outcome analyzed with both multiadjusted logistic regression and time-to-event based Cox regression

MAKE subcomponents	MAKE/Total	Logistic OR (95% CI)	Cox HR (95% CI)
A1M			
Progressive CKD	341/1486	1.44 (1.11–1.87)^a^	1.16 (1.06–1.27)^a^
Rapid eGFR decline	232/1377	1.64 (1.29–2.09)^a^	n.a.
KRT	10/1155	1.61 (1.11–2.35)^a^	2.75 (1.66–4.57)^a^
Kidney death	9/1154	3.20 (1.93–5.32)^a^	1.24 (0.88–1.72)
B2M			
Progressive CKD	342/1492	1.06 (0.87–1.30)	1.10 (1.00–1.22)^a^
Rapid eGFR decline	233/1383	1.29 (1.08–1.54)^a^	n.a.
KRT	10/1160	2.76 (0.94–8.12)	1.46 (1.02–2.11)^a^
Kidney death	9/1159	2.26 (1.20–4.27)^a^	1.27 (0.77–2.07)

A1M, alpha-1-microglobulin; B2M, beta-2-microglobulin; CI, confidence interval; CKD, chronic kidney disease; eGFR, estimated glomerular filtration rate; HR, hazard ratio; KRT, kidney replacement therapy; MAKE, major adverse kidney events; n.a., not available; OR, odds ratio.

For Cox regression analyses, the rapid eGFR decline outcome was not included in the MAKE composite outcome because it is not possible to measure or estimate time-to-event for this outcome.

a *P* < 0.05.

**Figure 3:**
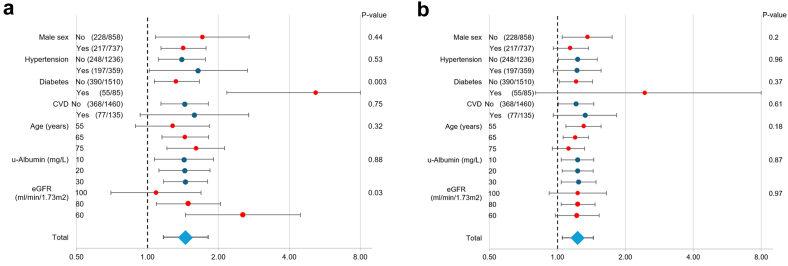
Effect of various moderators on the multiadjusted odds ratio for major adverse kidney events per 1 SD higher levels of (panel A) alpha-1-microglobulin and (panel B) B2M in the urine. Data describe the odds ratios (95% confidence interval) for major adverse kidney events after adjusting for age, sex, antihypertensive medication, systolic blood pressure, body mass index, smoking status, diabetes mellitus, CVD, urine creatinine, urine albumin, and eGFR in various subgroups. Data are exploratory and not adjusted for multiple testing. For continuous moderators, risk was estimated for the 50th, 75th, and 90th percentile of the covariate (or 50th, 25th, and 10th percentiles when lower values are associated with increased risk [eGFR]). CVD, cardiovascular disease; eGFR, estimated glomerular filtration rate; u-Albumin, urinary albumin.

**Figure 4:**
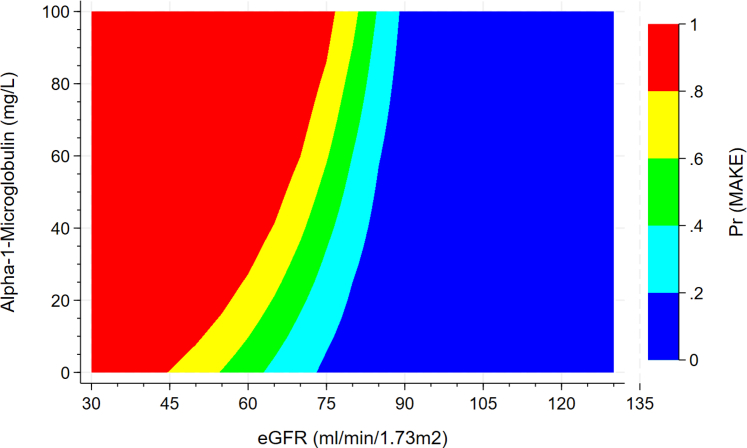
Two-way sensitivity analysis of predicted probability (Pr) of MAKE over commonly observed values of urine alpha-1-microglobulin level versus eGFR levels. The model was adjusted for demographics and traditional chronic kidney disease risk factors, including urinary albumin-to-creatinine ratio. eGFR, estimated glomerular filtration rate; MAKE, major adverse kidney events.

We evaluated the fully adjusted models across key subgroups (Figure 3), and associations of A1M and B2M with MAKE appeared similar across strata of age, sex, hypertension, CVD, and albuminuria. However, there was a significant interaction for A1M with diabetes (*P* interaction = 0.003) with stronger association of A1M with MAKE among participants with diabetes mellitus compared with those without diabetes. Furthermore, a significant interaction between A1M and eGFR (*P* interaction = 0.03) indicated weaker association for A1M with MAKE at higher eGFR levels. This was further explored in a 2-way sensitivity analysis over a range of commonly observed values for these 2 variables (Figure 4). In this analysis, A1M concentrations appeared to particularly influence MAKE risk among persons with eGFR between 45 to 75 ml/min per 1.73 m^2^, a range comprising approximately one-tenth of the study population. For example, the predicted multiadjusted risk for MAKE for a person with eGFR of 75 ml/min per 1.73 m^2^ was estimated to be very low (0.0–0.2) if the A1M was in the normal range, whereas the risk would be medium (0.4–0.6) if the person had elevated A1M levels > 50 mg/l. Urine B2M showed similar patterns (Supplementary Figure S2).

## Discussion

In this study of community-dwelling adults in Norway, we found that higher urine A1M and B2M, but not CysC, concentrations were associated with higher risks of MAKE. These associations remained statistically significant after adjustment for eGFR, uACR , and traditional kidney risk factors; and support the use of proximal tubule reabsorption biomarkers for capturing risk of earlier CKD progression.

Strong, independent, and consistent associations between urinary A1M and cardiovascular and kidney outcomes have been demonstrated in several studies. Urinary A1M associates with allograft failure in stable kidney transplant recipients,^7^ acute kidney injury in treated high risk hypertensive participants,^19^ and CVD and mortality in a hypertensive nondiabetic CKD cohort.^20^ Several studies have shown that starting tenofovir, a treatment for HIV, which is known to specifically damage the proximal tubules, is associated with longitudinal increases in A1M urine concentration,^21^ and higher A1M is known to be associated with faster eGFR decline in HIV.^22^ However, no significant association of A1M was found with CKD progression among persons with diabetic kidney disease,^23^ or in a cohort of participants with hypertension and high CVD risk.^24^ Studies evaluating persons in the general population are more scarce. Urinary A1M was significantly associated with incident CKD in 1 general population-based study among those without CKD or diabetes (ARIC), but results were not confirmed in 2 other similar cohorts (MESA, REGARDS).^8^ Our results support and extend these findings as we demonstrated a strong association between A1M and MAKE in the general population—findings that were above and beyond albuminuria and eGFR.

The precise pathophysiological mechanisms underlying these associations are not yet fully understood, though they likely center on the function of proximal tubule transport proteins such as megalin and cubulin. These are critical proximal tubule transport proteins that can bind and reabsorb a wide range of carrier proteins, hormones, enzymes, lipoproteins, and immune- or stress-related proteins, including A1M and B2M.^25^ Megalin expression and function can be reduced by hyperglycemia,^26^ and severe albuminuria can saturate megalin receptors leading to reduced reabsorption of our biomarkers. However, we adjusted for diabetes status in our analyses, and our participants had normal or only moderately increased albumin excretion. Aminoglycosides and cisplatin can cause intermittent or permanent tubular damage, but we are not aware of drugs with a direct inhibitory effect on proximal tubule reabsorption.

Although less is known about the reabsorption of CysC, previous studies indicate that the same megalin mechanisms may apply,^27^ and higher urine concentrations of CysC have been found in persons with CKD in a few cross-sectional studies.^28^ However, few if any studies, including ours, have demonstrated that higher urine levels are associated with future adverse outcomes.^22^^,^^29^^,^^30^ The reasons why A1M and B2M, but not CysC, are associated with MAKE are unknown, and a range of underlying mechanisms, including differences in filtered load, receptor-binding dynamics, and intracellular handling, could be involved. Furthermore, the reported associations between urine B2M and future CVD and kidney disease have been inconsistent.^20^^,^^24^^,^^31^ A possible explanation might be that the protein is unstable in urine with substantial degradation after a few hours.^32^ Our study is one of the first to demonstrate a significant association of urine B2M with MAKE in a large cohort, and meticulous attention to preanalytical quality control and rapid deep freezing of fresh urine specimens may explain why we found strong associations of B2M with MAKE that were comparable to those with higher A1M.^33^

A1M is excreted in relatively high concentrations and can be reliably measured with very good accuracy using standard clinical chemistry methods available in most laboratories. Our findings support the general hypothesis that decreased proximal tubule function is associated with CKD progression, and urine A1M could add important information, especially for patients with eGFR of 45–75 ml/min per 1.73 m^2^. In addition, knowledge of tubule dysfunction and damage could be useful for monitoring treatments with known or suspected nephrotoxicity, including anti-HIV drugs, aminoglycosides, cytotoxic drugs, and others. There may be utility to screen for environmental toxicity in populations, because low-grade exposure of heavy metals and other toxins are known to be associated with reduced kidney function, and proximal tubule damage is a major mechanism.^34^^,^^35^ Furthermore, even when fibrosis and tubule atrophy are observed on biopsy, for example, in chronic allograft nephropathy, repeat biopsies are seldom done, and urine markers of tubule reabsorption could provide a way to monitor tubule health longitudinally. Therefore, quantification of A1M in the urine could have several clinical implications because it suggests a new prognostically useful dimension of kidney health for clinicians and a reliable measurement is already clinically available. Understanding the cross-talk between glomeruli and tubules is essential for elucidating pathophysiology and identifying new treatments.^36^ B2M may provide similar opportunities; however, because A1M is measured by nephelometry, it is more readily available in most clinical chemistry laboratories.

The main strength of this study is its use of the well-characterized community-living HUNT study with its high-quality biological samples, including freezing urine samples within 2 hours. Other key strengths include the availability of several biomarkers of proximal tubule resorption concurrently; detailed ascertainment of key covariates; long-term follow-up for MAKE events; and stability of results over MAKE subcomponents, different regression methods, and other outcomes. However, the study has important limitations. Urine biomarkers were measured at a single time point, so within-individual variability is uncertain. The study had limited power for small subgroups (e.g., diabetes), and the number of participants with abnormally high biomarker levels was relatively low, contributing to wide CIs. The MAKE composite outcome was driven primarily by eGFR-based events. The associations with the small number of hard clinical end points (KRT or kidney death) should therefore be interpreted with caution. Considering that this is a hypothesis-generating study rather than a confirmatory analysis, we did not correct for multiple testing, and the reported associations should be interpreted with caution. We included a predominantly White population with largely preserved kidney health, which limits generalization. Studies in other regions and populations are needed to confirm these findings.

In this community-based study, we demonstrate that biomarkers reflecting diminished proximal tubule reabsorption (A1M and B2M) are significantly associated with the risk of MAKE, especially the risk of earlier CKD progression, independent of albuminuria, eGFR, and other CKD risk factors. Urine A1M, which is already available for clinical testing, may give insight into an axis of kidney health beyond glomerular filtration and provide new opportunities for identifying CKD progression risk and drug toxicity monitoring.

## Disclosure

All the authors declared no competing interests.

## Supplementary Material

Supplementary File (PDF) **Figure S1.** Distribution of biomarker values by outcome (no-MAKE vs. MAKE). **Figure S2.** Levels of predicted probability of MAKE by urine beta-2-microglobulin versus eGFR after adjustment for demographics and traditional CKD risk factors, including uACR. **Table S1.** Comparison of baseline characteristics in the current study versus total HUNT-3. **Table S2.** Prevalence of abnormal high biomarker levels (> 97.5th percentile in healthy subjects) by relevant subgroups of participants. **Table S3.** Sensitivity analyses displaying associations between tubular reabsorption biomarkers (A1M and B2M) and eGFR declines > 30% and 40%, respectively, from baseline. **Table S4.** Sensitivity analyses displaying associations between tubular reabsorption biomarkers (A1M and B2M) indexed to urine creatinine or osmolality and MAKE. **Table S5.** Sensitivity analysis comparing multiple imputations of A1M values below limit of detection (LOD) versus replacing all values < LOD with the LOD cutoff (5.6 mg/l) for the association between A1M and MAKE.

## Footnotes

Supplementary File (PDF)
**Figure S1.** Distribution of biomarker values by outcome (no-MAKE vs. MAKE). **Figure S2.** Levels of predicted probability of MAKE by urine beta-2-microglobulin versus eGFR after adjustment for demographics and traditional CKD risk factors, including uACR. **Table S1.** Comparison of baseline characteristics in the current study versus total HUNT-3. **Table S2.** Prevalence of abnormal high biomarker levels (> 97.5th percentile in healthy subjects) by relevant subgroups of participants. **Table S3.** Sensitivity analyses displaying associations between tubular reabsorption biomarkers (A1M and B2M) and eGFR declines > 30% and 40%, respectively, from baseline. **Table S4.** Sensitivity analyses displaying associations between tubular reabsorption biomarkers (A1M and B2M) indexed to urine creatinine or osmolality and MAKE. **Table S5.** Sensitivity analysis comparing multiple imputations of A1M values below limit of detection (LOD) versus replacing all values < LOD with the LOD cutoff (5.6 mg/l) for the association between A1M and MAKE.
