# Supplementary material for: Proximal Tubule Reabsorption and CKD Progression in the General Population
Source: Kidney Int Rep. 2026 Jan 29;11(4):103801. doi: 10.1016/j.ekir.2026.103801 (PMC12966674; doi:10.1016/j.ekir.2026.103801)

Supplementary Table S1: Comparison of baseline characteristics in the current study versus total HUNT-3

	Current study (N=1595)	Total HUNT-3 (N=50583)
Age (years)	52.8 (15.4)	53.1 (16.1)
Male	46.2 %	45.3 %
Never-smoker	41.4 %	43.6 %
Former-smoker	29.5 %	28.0 %
Current-smoker	18.9 %	19.2 %
BMI (kg/m2)	27.0 (4.3)	27.2 (4.4)
sBP (mmHg)	130.8 (17.5)	130.4 (18.6)
dBP (mmHg)	74.1 (11.8)	73.2 (11.2)
Treated HT	22.5 %	22.5 %
DM	5.3 %	4.6 %
CVD	9.0 %	7.8 %
Chol (mmol/L)	5.5 (1.1)	5.5 (1.1)
ACR (mg/mmol)	1.34 (1.00-1.86)	1.29 (0.97-1.76))

eGFR (ml/min/1.73m2)	92.1 (21.7)	95.3 (18.8)

Supplementary Table S2: Prevalence of abnormal high biomarker levels (>97.5th percentile in healthy subjects) by relevant subgroups of participants.

	Healthy subjects	Random subcohort	Total cohort	No MAKE	MAKE	eGFR <60 ml/min/1.73m2
Alpha-1-Microglobulin (>22.1 mg/L)	2.6%	5.5%	8.3%	5.0%	17.5%	24.1%
Beta-2-Microglobulin (>2.1 mg/L)	2.4%	3.0%	4.2%	2.6%	8.5%	13.1%
Cystatin C (>2.0 mg/L)	2.9%	3.4%	3.7%	3.3%	4.9%	7.5%

Supplementary Table S3: Sensitivity analyses displaying associations between tubular reabsorption biomarkers (A1M and B2M) and eGFR declines of more than 30% and 40%, respectively, from baseline.

	MAKE/Total	Model1	Model 2	Model 3
A1M				
>30% eGFR decline	229/1595	**1.92 (1.25-2.95)**	**1.39 (1.07-1.80)**	1.29 (0.97-1.74)
>40% eGFR decline	161/1595	**1.92 (1.26-2.94)**	**1.54 (1.18-2.02)**	**1.44 (1.00-2.08)**
B2M				
>30% eGFR decline	229/1595	**1.45 (1.21-1.74)**	**1.29 (1.08-1.53)**	**1.24 (1.00-1.53)**
>40% eGFR decline	161/1595	**1.49 (1.27-1.76)**	**1.31 (1.11-1.55)**	1.21 (0.99-1.50)

Note: Data are odds ratios (95% CI) per 1 SD increase of biomarker in the urine. Model 1 is adjusted for urine creatinine. Model 2 is additionally adjusted for age, sex, systolic BP, antihypertensive medication, BMI, smoking, diabetes and a history of CVD. Model 3 is additionally adjusted for urine albumin and eGFR. P<0.05 marked in bold.

Supplementary Table S4: Sensitivity analyses displaying associations between tubular reabsorption biomarkers (A1M and B2M) indexed to urine creatinine or osmolality and MAKE.

	Model1	Model 2	Model 3
A1M			
Indexed to u-creatinine	**1.60 (1.38-1.85)**	**1.38 (1.17-1.62)**	**1.28 (1.00-1.65)**
Indexed to u-osmolality	**2.84 (1.82-4.43)**	**2.30 (1.65-3.21)**	**1.74 (1.17-2.61)**
B2M			
Indexed to u-creatinine	**1.45 (1.21-1.74)**	**1.40 (1.19-1.65)**	**1.22 (1.02-1.47)**
Indexed to u-osmolality	**1.78 (1.48-2.14)**	**1.65 (1.36-2.01)**	**1.31 (1.02-1.68)**

Note: Data are odds ratios (95% CI) per 1 SD increase of biomarker in the urine. Model 1 is based on biomarker indexed to urine creatinine or osmolality. Model 2 is additionally adjusted for age, sex, systolic BP, antihypertensive medication, BMI, smoking, diabetes and a history of CVD. Model 3 is additionally adjusted for urine ACR and eGFR. P<0.05 marked in bold.

Supplementary Table S5: Sensitivity analysis comparing multiple imputation of A1M values below limit of detection (LOD) versus replacing all values <LOD with the LOD cutoff (5.6 mg/L) for the association between A1M and MAKE.

	Model1	Model 2	Model 3
A1M <LOD Imputed	**1.58 (1.24-2.02)**	**1.47 (1.18-1.83)**	1.37 (0.98-1.91)
A1M <LOD = LOD	**1.74 (1.24-2.42)**	**1.53 (1.24-1.90)**	**1.45 (1.17-1.81)**

Note: Data is odds ratio (95% CI) for MAKE based on 20 imputed datasets using a truncated regression imputation method. Model 1 is based on biomarker indexed to urine creatinine or osmolality. Model 2 is additionally adjusted for age, sex, systolic BP, antihypertensive medication, BMI, smoking, diabetes and a history of CVD. Model 3 is additionally adjusted for urine ACR and eGFR. P<0.05 marked in bold.

Supplementary Figure S1. Distribution of biomarker values by outcome (no MAKE vs MAKE)

Panel A

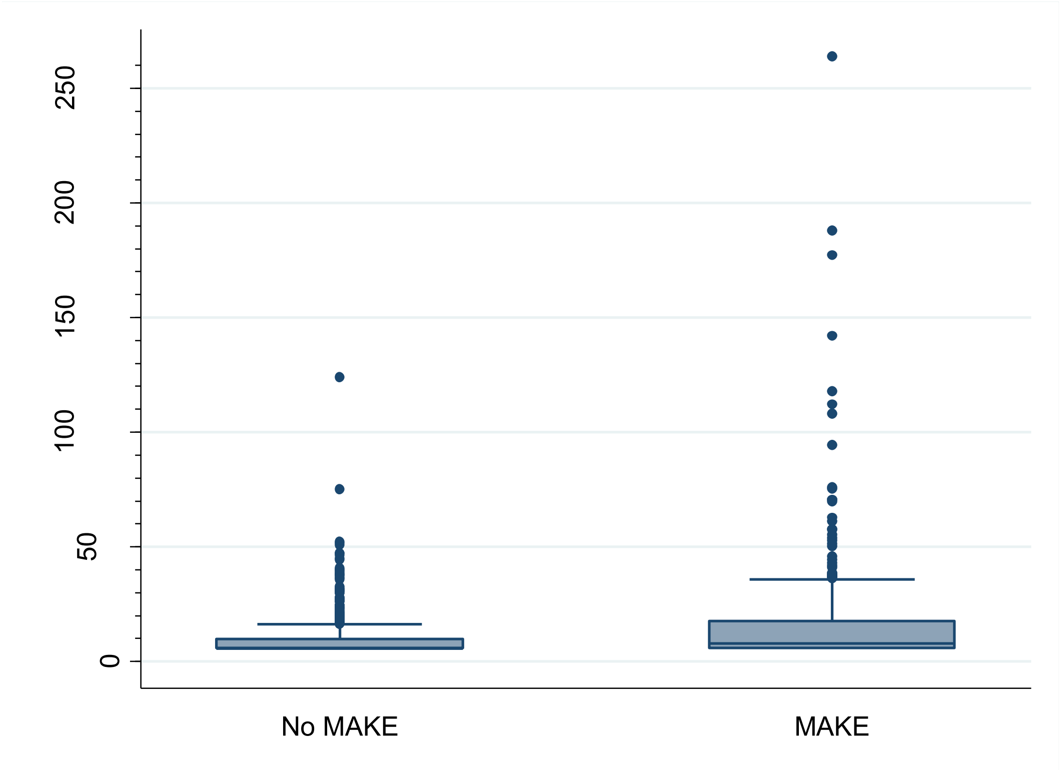

Panel B

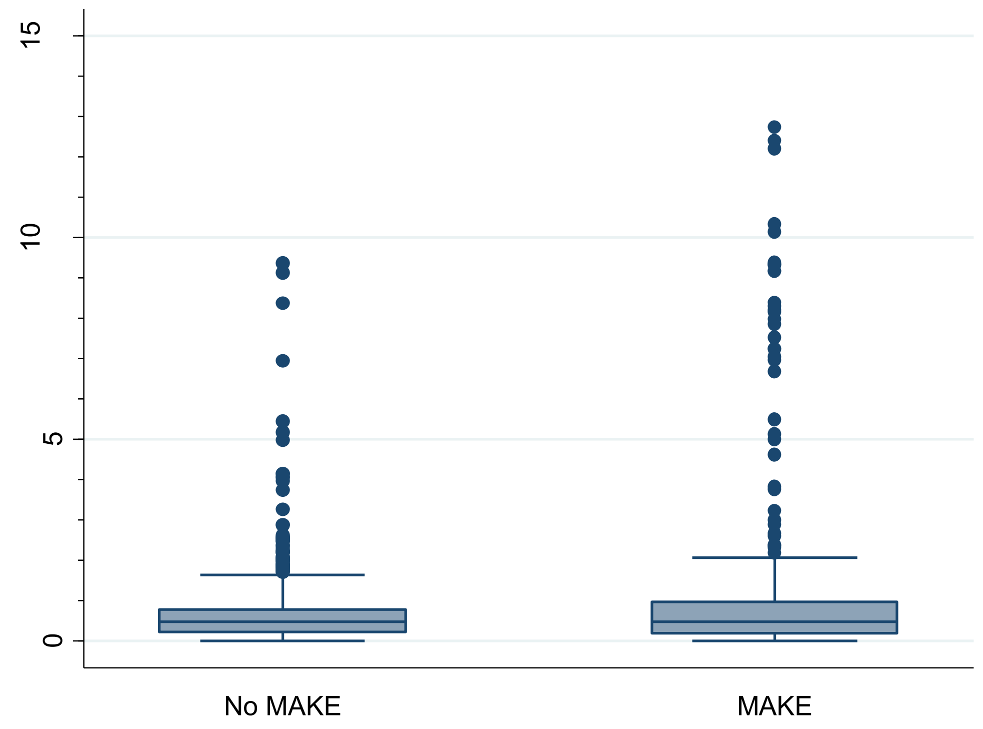

Panel C

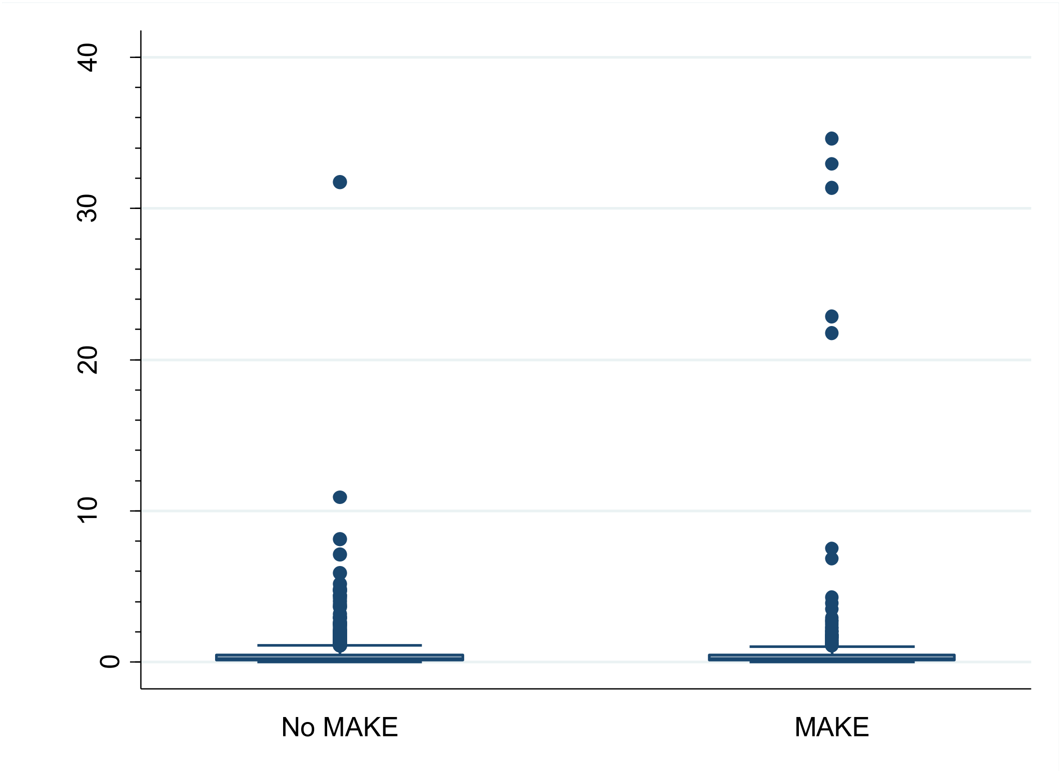

Supplementary Figure S2: Levels of predicted probability of MAKE by urine beta-2-microglobulin versus eGFR after adjustment for demographics and traditional CKD risk factors including u-ACR.

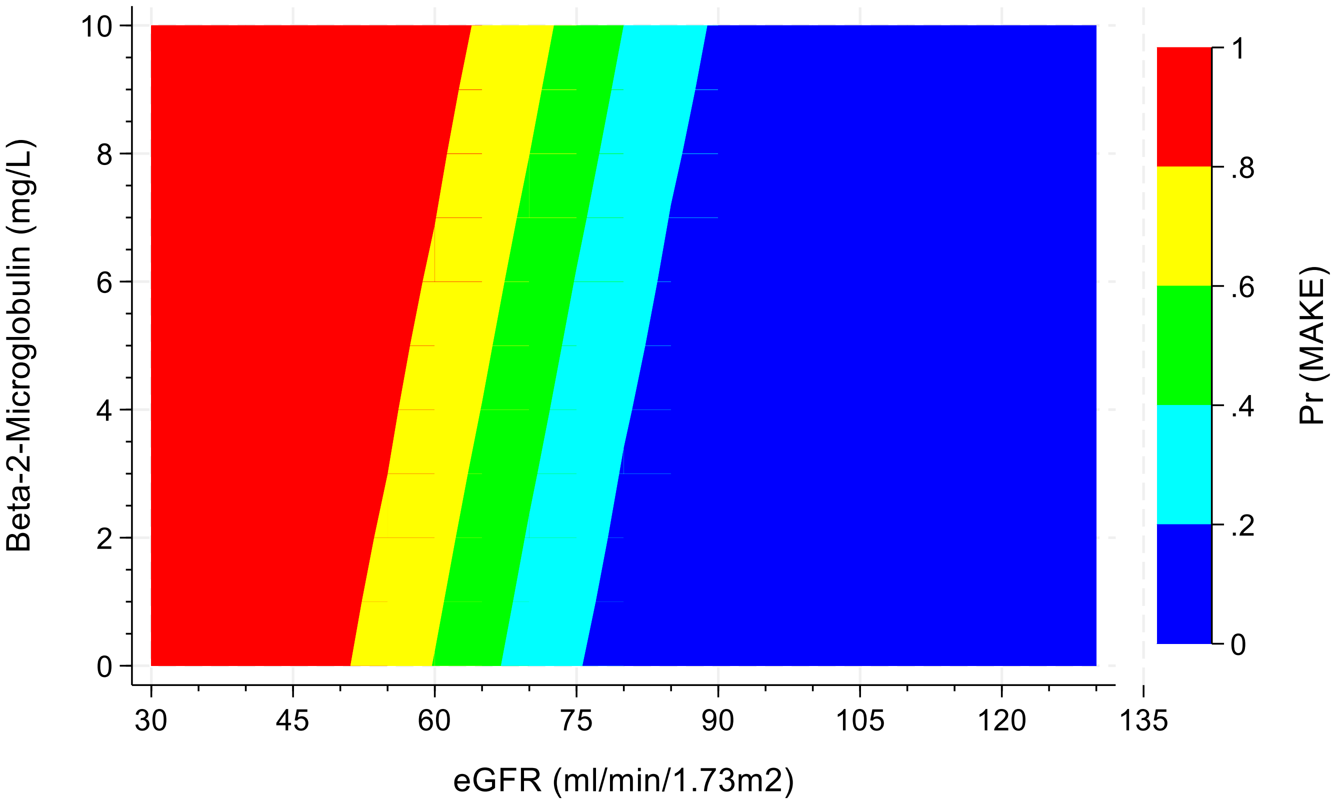

Supplement: Supplementary File (PDF) — Figure S1. Distribution of biomarker values by outcome (no-MAKE vs. MAKE). Figure S2. Levels of predicted probability of MAKE by urine beta-2-microglobulin versus eGFR after adjustment for demographics and traditional CKD risk factors, including uACR. Table S1. Comparison of baseline characteristics in the current study versus total HUNT-3. Table S2. Prevalence of abnormal high biomarker levels (> 97.5th percentile in healthy subjects) by relevant subgroups of participants. Table S3. Sensitivity analyses displaying associations between tubular reabsorption biomarkers (A1M and B2M) and eGFR declines > 30% and 40%, respectively, from baseline. Table S4. Sensitivity analyses displaying associations between tubular reabsorption biomarkers (A1M and B2M) indexed to urine creatinine or osmolality and MAKE. Table S5. Sensitivity analysis comparing multiple imputations of A1M values below limit of detection (LOD) versus replacing all values < LOD with the LOD cutoff (5.6 mg/l) for the association between A1M and MAKE. [file mmc1.pdf]
